# Orexinergic System in Neurodegenerative Diseases

**DOI:** 10.3389/fnagi.2021.713201

**Published:** 2021-08-17

**Authors:** Qinqin Wang, Fei Cao, Yili Wu

**Affiliations:** ^1^Shandong Collaborative Innovation Center for Diagnosis, Treatment & Behavioral Interventions of Mental Disorders, Institute of Mental Health, Jining Medical University, Jining, China; ^2^Shandong Key Laboratory of Behavioral Medicine, School of Mental Health, Jining Medical University, Jining, China; ^3^Key Laboratory of Alzheimer’s Disease of Zhejiang Province, Institute of Aging, School of Mental Health and The Affiliated Kangning Hospital, Wenzhou Medical University, Wenzhou, China; ^4^Oujiang Laboratory, Wenzhou, China

**Keywords:** orexinergic system, Alzheimer’s disease, Parkinson’s disease, Huntington’s disease, multiple sclerosis

## Abstract

Orexinergic system consisting of orexins and orexin receptors plays an essential role in regulating sleep–wake states, whereas sleep disruption is a common symptom of a number of neurodegenerative diseases. Emerging evidence reveals that the orexinergic system is disturbed in various neurodegenerative diseases, including Alzheimer’s disease (AD), Parkinson’s disease (PD), Huntington’s disease (HD), and multiple sclerosis (MS), whereas the dysregulation of orexins and/or orexin receptors contributes to the pathogenesis of these diseases. In this review, we summarized advanced knowledge of the orexinergic system and its role in sleep, and reviewed the dysregulation of the orexinergic system and its role in the pathogenesis of AD, PD, HD, and MS. Moreover, the therapeutic potential of targeting the orexinergic system for the treatment of these diseases was discussed.

## Introduction

Orexinergic system is essential for the maintenance of various physiological processes including sleep–wake states (Gamble et al., 2019; Yukitake et al., 2019; Zhang et al., 2019). Emerging evidence reveals that the orexinergic system is disturbed in various neurodegenerative diseases, such as Alzheimer’s disease (AD), Parkinson’s disease (PD), Huntington’s disease (HD), and multiple sclerosis (MS), whereas the dysregulation of the orexinergic system plays a pivotal role in the pathogenesis of these diseases (Petersen et al., 2005; Asai et al., 2009; Fatemi et al., 2016; Mander et al., 2016; Liguori, 2017). To gain a better understanding of the therapeutic potential of targeting the orexinergic system for the treatment of these diseases, we extensively reviewed the characteristics of the orexinergic system, its function in sleep–wake states, and its role in the pathogenesis of the neurodegenerative diseases and underlying mechanisms.

## Overview of Orexinergic System

Orexinergic system consists of orexins and their receptors. Orexin, also named as hypocretin, includes two isoforms, namely, orexin A (OXA) or hypocretin-1 (HCRT-1) and orexin B (OXB) or hypocretin-2 (HCRT-2) (de Lecea et al., 1998; Sakurai et al., 1998; Soya and Sakurai, 2020). OXA and OXB are generated from the same prepro-orexin precursor *via* differential hydrolysis (Gotter et al., 2012). OXA is a ∼3.5 kDa peptide with 33 amino acids, whereas OXB is a ∼2.9 kDa peptide with 28 amino acids. Both of them are highly conserved in mammalian species (Sakurai et al., 1998; Dyer et al., 1999; Spinazzi et al., 2006). Orexins are mainly expressed in lateral hypothalamic neurons (Wang et al., 2018). Orexin receptors, including orexin 1 receptor (OX1R) and orexin 2 receptor (OX2R), are G-protein-coupled receptors (GPCRs) (de Lecea et al., 1998; Sakurai et al., 1998; Soya and Sakurai, 2020). OX1R is mainly expressed in ventromedial hypothalamic nucleus, dorsal raphe, locus coeruleus, and hippocampus, whereas OX2R is highly expressed in nucleus accumbens, anterior pretectal nucleus, and cerebral cortex (Trivedi et al., 1998; Soya et al., 2013). Both of them are highly conserved in mammals, for example, human OX1R and OX2R share 94 and 95% identity with rat OX1R and OX2R, respectively. Human OX1R and OX2R consist of 425 and 444 amino acids, respectively, and they share approximately 64% sequence identity (Spinazzi et al., 2006). OXA binds to both OX1R and OX2R with similar affinity (Gotter et al., 2012; Wang et al., 2018), whereas OXB prefers to bind to OX2R (Gotter et al., 2012; Wang et al., 2018).

The main function of the orexinergic system is to regulate sleep–wake states (Adamantidis et al., 2007; Gamble et al., 2019; Yukitake et al., 2019; Zhang et al., 2019). Orexin deficiency is the proximal cause of human narcolepsy with cataplexy, which has been comprehensively studied and reviewed (Bassetti et al., 2019; Nepovimova et al., 2019). Consistently, OX2R agonist YNT-185 ameliorates narcolepsy symptoms in a mouse model of narcolepsy-cataplexy (Irukayama-Tomobe et al., 2017). Photostimulation of orexin neurons induces the transition of sleep to wake in mice, indicating that orexin neurons play a key role in promoting sleep-to-wake transition (Adamantidis et al., 2007). Activation of orexin neurons leads to a marked increase of wakefulness time and a reduction of sleep time including both rapid eye movement (REM) sleep and non-REM (NREM) sleep in mice (Sasaki et al., 2011). Consistently, OX2R agonist YNT-185 markedly increases wakefulness time in mice (Irukayama-Tomobe et al., 2017).

## Orexinergic System in Neurodegenerative Diseases

### Orexinergic System in AD

AD, the most common neurodegenerative disease, is characterized by the clinical manifestations such as progressive memory loss, cognitive deficits, and sleep disruption. Neuritic plaques, neurofibrillary tangles, and neuronal loss are the neuropathological hallmarks of AD. β-Amyloid (Aβ) and hyperphosphorylated Tau are the core components of neuritic plaques and neurofibrillary tangles, respectively (Lane et al., 2018; Qiu et al., 2019).

Although studies indicate that the orexin levels in cerebrospinal fluid (CSF) are associated with circadian alteration in AD and the OXA levels are positively correlated with the cognitive function in AD (Liguori et al., 2020; Shimizu et al., 2020), the dysregulation of the orexinergic system in AD remains inconclusive. For example, a significant reduction of OXA positive neurons and the levels of OXA were observed in patients with AD (Fronczek et al., 2012), whereas both Slats et al. and Liguori et al. reported that there was no difference of OXA in CSF of patients with AD and healthy controls (Slats et al., 2012; Liguori et al., 2014). In addition, Gabelle et al. reported that OXA was increased in AD (Gabelle et al., 2017). The inconsistent results might result from the different methods and sample sizes. For example, studies by Fronczek et al. and Gabelle et al. were performed in postmortem ventricular CSF and the stored CSF samples, respectively (Fronczek et al., 2012; Gabelle et al., 2017), whereas the participants were awaked in studies performed by Slats et al. and Liguori et al., respectively (Slats et al., 2012; Liguori et al., 2014). Moreover, increased orexin along with more fragmented sleep was observed in patients with AD with neuropsychiatric symptoms compared with that in patients with AD without neuropsychiatric symptoms (Liguori et al., 2018).

**FIGURE 1:**
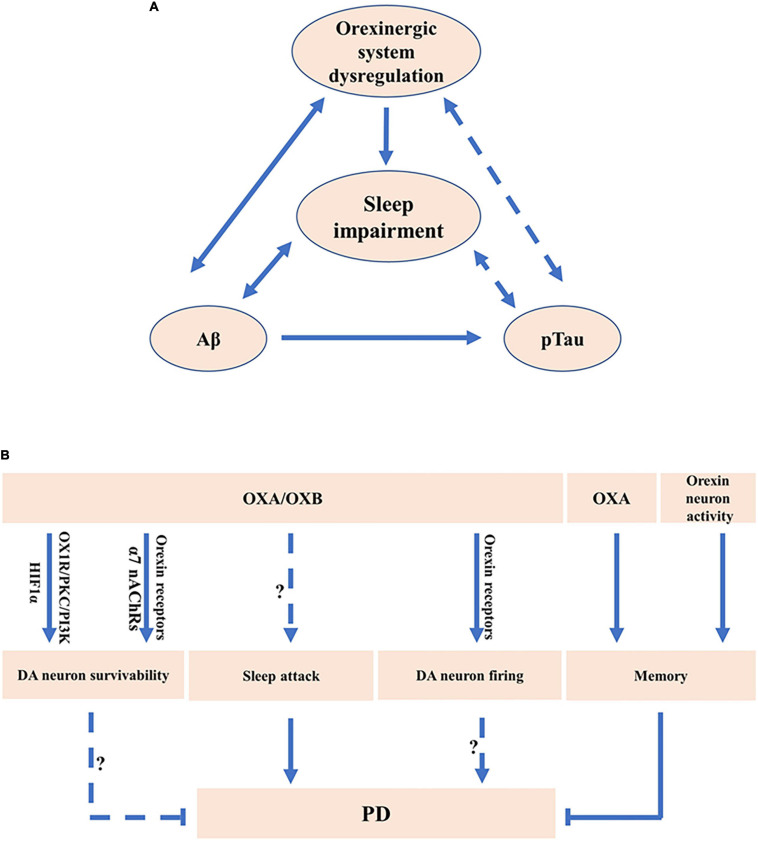
The potential roles of orexinergic system in Alzheimer’s disease (AD) and Parkinson’s disease (PD). **(A)** The potential roles of orexinergic system in AD. Orexinergic system is involved in the regulation of sleep–wake states. Sleep disruption facilitated Aβ accumulation in AD. Orexin A (OXA) is associated with Aβ. Evidence also suggests a potential relationship between Aβ levels and sleep–wake states. Moreover, OXA in cerebrospinal fluid (CSF) was correlated with pTau. The levels of pTau were correlated with stage 1 of NREM sleep. More experiments are needed to clarify the correlation between pTau and orexinergic system-related sleep dysfunction. **(B)** The potential roles of orexinergic system in PD. OXA exerted the neuroprotective roles in PD cell model *via* regulating HIF-1α or orexin 1 receptor (OX1R)/protein kinase C (PKC)/PI3-kinase (PI3K) signaling. Orexin 2 receptor (OX2R) and α7 nAChRs were involved in the promotion of the survivability of DA neurons regulated by orexin B (OXB). OXA treatment or enhancement of orexin neurons activity markedly decreased the memory impairment in PD mice. OXA and OXB significantly increased the rate of spontaneous firing of dopaminergic (DA) neurons *via* OX1R and OX2R. Sleep attack has been observed in PD. Orexinergic system was involved in sleep disorders, indicating the potential roles of orexinergic system in regulating PD-related sleep disorders.

Orexinergic system is implicated in Aβ pathology. First, the levels of OXA were correlated with the level of Aβ_42_ in patients with AD (Slats et al., 2012; Gabelle et al., 2017; Figure 1A). Importantly, orexin deficiency markedly decreased Aβ pathology in AD model mice, amyloid precursor protein/presenilin 1 (APP/PS1) transgenic mice (Roh et al., 2014). Moreover, sleep disruption, a common symptom of AD, facilitated Aβ accumulation, contributing to neurodegeneration in AD, which might be correlated with the alteration of the orexinergic system (Mander et al., 2016; Liguori, 2017; Figure 1A). Reduced Aβ_1__–__42_ was correlated with the decrease of REM sleep and sleep efficiency in AD, whereas the increase of OXA in CSF was correlated with the decrease of REM and sleep efficiency in AD (Liguori et al., 2014). The diurnal variation of Aβ levels in interstitial fluid (ISF) was observed in human. Consistently, the Aβ levels in ISF were positively correlated with the awaked time and negatively correlated with the asleep time in mice (Kang et al., 2009; Figure 1A). In addition, the rate of Aβ_1__–__40_ clearance was much faster in sleeping mice than that in awaked mice (Xie et al., 2013). Furthermore, the administration of orexin significantly increased the Aβ levels in ISF during the light phase and orexin receptors antagonist led to the inhibition of the diurnal fluctuation of Aβ in ISF of AD model mice (Kang et al., 2009). On the other hand, increased Aβ contributes to the dysregulation of the orexinergic system and sleep disruption. For example, the administration of Aβ_25__–__35_ markedly increased awaked time and reduced NREM sleep in AD mice (Liu et al., 2019; Figure 1A). Aβ_25__–__35_ significantly increased the level of OXA in AD mice (Liu et al., 2019; Figure 1A).

The orexinergic signaling is associated with Tau pathology in AD (Deuschle et al., 2014; Liguori et al., 2014; Liguori, 2017). The level of OXA in CSF was correlated with Tau and phosphorylated Tau (pTau) in patients with AD (Deuschle et al., 2014; Figure 1A). Consistently, the level of OXA in CSF exhibited an evidently positive correlation with Tau and pTau in patients with moderate to severe AD (Liguori et al., 2014), and even in control subjects (Shimizu et al., 2020). Importantly, the OXA downregulation dramatically decreased the levels of Tau and pTau induced by Aβ_25__–__35_ *in vitro* (Liu et al., 2019; Figure 1A). Moreover, studies show that the levels of pTau were correlated with stage 1 of NREM sleep in AD (Liguori et al., 2014; Figure 1A). It indicates that the role of orexinergic signaling in Tau pathology may be related to sleep dysfunction.

Dysregulation of orexinergic signaling may be associated with neurodegeneration and neuronal loss in AD. First, AD was primarily associated with the loss of basal forebrain cholinergic neurons (Zajo et al., 2016). Basal forebrain was one of the major projection targets of orexin neurons (Li and de Lecea, 2020). Orexin receptors were expressed in the basal forebrain (Li and de Lecea, 2020), while orexin neurons directly communicated with the cholinergic neurons in the basal forebrain through synapses (Li and de Lecea, 2020). Moreover, the administration of OXA in basal forebrain ameliorated distracter-induced attention deficiency in rats (Zajo et al., 2016). These results indicate that the orexinergic system may be involved in AD pathology by regulating the cholinergic pathway in the basal forebrain.

### Orexinergic System in PD

PD is characterized by the selective loss of dopaminergic (DA) neurons in the substantia nigra (SN) and the formation of Lewy body (Kalia and Lang, 2015; Reich and Savitt, 2019). The clinical symptoms include both motor symptoms such as resting tremor and bradykinesia and non-motor symptoms such as pain, sleep disorder, and cognition dysfunction (Kalia and Lang, 2015).

The dysregulation of the orexinergic system in PD is inconclusive as the number of studies is limited. First, it was reported that the level of OXA was significantly decreased in ventricular CSF of patients with advanced PD compared with controls (Drouot et al., 2003). Consistently, the number of orexin neurons was markedly decreased in patients with PD compared with that in controls (Fronczek et al., 2007). However, the level of OXA was within the normal range in CSF obtained from patients with PD by lumbar puncture (Yasui et al., 2006).

Orexinergic system is implicated in the neuroprotective effect on DA neurons (Cui et al., 2010; Feng et al., 2014; Guerreiro et al., 2015; Hadadianpour et al., 2017; Pasban-Aliabadi et al., 2017; Liu M. F. et al., 2018). The number of OXA positive neurons tended to be decreased in hypothalamus of PD model rats (Cui et al., 2010). OXA significantly ameliorated 1-methyl-4-phenylpyridinium (MPP^+^)-induced injury in SH-SY5Y cells, which was mediated by hypoxia inducible factor-1 alpha (HIF-1α) (Feng et al., 2014; Figure 1B). Moreover, OXA significantly attenuated 6-hydroxydopamine (6-OHDA)-induced cell toxicity in SH-SY5Y cells, which was mediated by OX1R/protein kinase C (PKC)/PI3-kinase (PI3K) signaling (Pasban-Aliabadi et al., 2017; Figure 1B). In addition, OXA significantly attenuated 1-methyl-4-phenyl-1, 2, 3, 6-tetrahydropyridine (MPTP)-induced DA neuron loss in the SN of mice, contributing to the improvement of cognitive and motor ability (Liu M. F. et al., 2018), whereas OX1R antagonist SB334867 could inhibit the protective effect (Liu M. F. et al., 2018; Figure 1B). Consistently, OXA significantly ameliorated 6-OHDA-induced impairments of locomotor function, sensorimotor function, and muscle tone in rats (Hadadianpour et al., 2017). Furthermore, OXB increased the survivability of DA neurons mediated by OX2R but not by OX1R, whereas nicotine markedly improved the neuroprotective effects of OXB on DA neuron *via* the activation of α7 nicotinic acetylcholine receptors (α7 nAChRs) (Guerreiro et al., 2015; Figure 1B). The above evidence indicates that orexins and orexin receptors are involved in the protective effect on DA neurons in PD models.

Sleep disruption is a common symptom in PD (Asai et al., 2009; Nie et al., 2019). Most of these patients suffer from excessive sleepiness during daytime and some of them present the sleep attack (Tracik and Ebersbach, 2001; Brodsky et al., 2003; Asai et al., 2009). Some characteristics of sleep attack in patients with PD are similar to narcolepsy symptoms (Arnulf et al., 2000; Asai et al., 2009). Dopamine agonists were one of the major factors influencing the emergence of sleep attack, suggesting the close correlation between DA system and sleep attack in PD (Paus et al., 2003; Figure 1B). Electrophysiological analysis revealed that the administration of OXA and OXB observably enhanced the rate of spontaneous firing of DA neurons in the SN of rats and inhibition of OX1R and OX2R significantly decreased the firing rate of DA neurons in the SN, implying the roles of the orexinergic pathway in regulating DA system (Liu C. et al., 2018; Figure 1B). Moreover, OXA application in the hippocampus or chemogenetic activation of orexin neurons significantly ameliorated the memory impairment in A53T transgenic mice, a PD model mice (Stanojlovic et al., 2019; Figure 1B). It indicates that the orexinergic system plays a key role in sleep disruption and memory defects in patients with PD.

### Orexinergic System in HD

**FIGURE 2:**
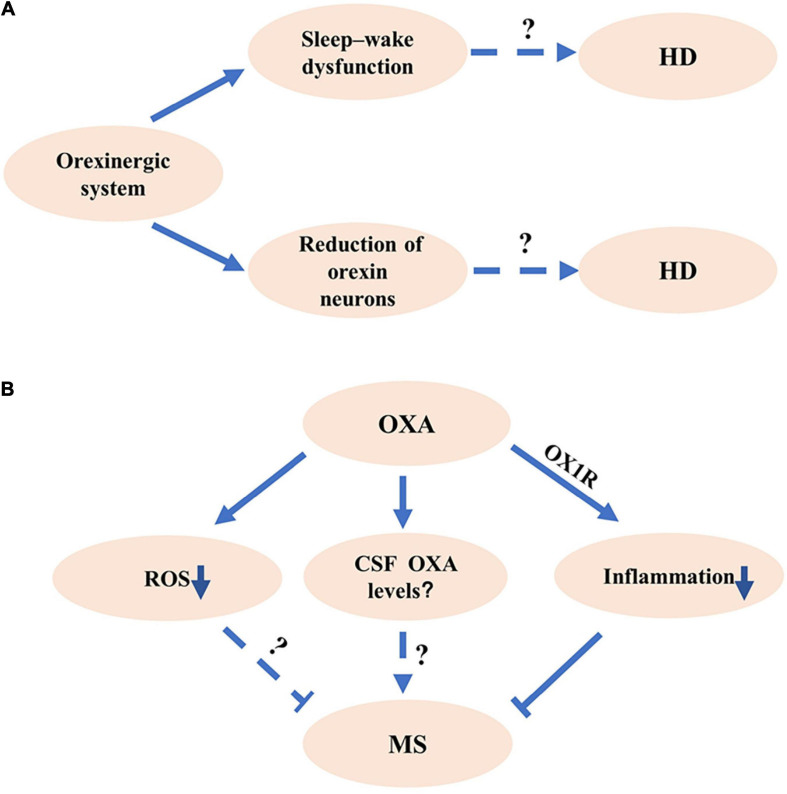
The potential roles of orexinergic system in Huntington’s disease (HD) and multiple sclerosis (MS). **(A)** The potential roles of orexinergic system in HD. Studies show that the number of OX neurons decreased in HD. Abnormal sleep existed in HD. The details of orexinergic signaling in HD need to be further investigated. **(B)** The potential roles of orexinergic system in MS. The OXA/OX1R signaling significantly decreased the inflammation and pathological processes in EAE mice. Moreover, OXA could markedly reduce the reactive oxygen species (ROS) and microglial activation, indicating that the orexinergic system might involve in MS pathology by regulating ROS and microglial activation. More experiments are needed to determine the relationship between orexin levels and MS pathology at different courses.

HD, a kind of polyglutamine disorder described first by George Huntington, presents autosomal dominant inheritance and is characterized by a progressive decline of cognition, motor, and behavioral functions (Wyant et al., 2017; Ghosh and Tabrizi, 2018; Croce and Yamamoto, 2019). Given that the molecular mechanisms underlying HD pathogenesis remain unclear, there is no effective treatment for HD (Ghosh and Tabrizi, 2018). Orexinergic system is involved in the pathogenesis of HD. A significant reduction of orexin neurons in the LH and hypothalamus was observed in patients with HD compared with that in controls (Petersen et al., 2005; Gabery et al., 2010; Figure 2A). Consistently, a significant decrease of OXA and OXB was observed in HD model mice (Petersen et al., 2005; Figure 2A).

The abnormal sleep characteristics were observed in HD model mice, including a shorter slow-wave sleep (SWS) duration, more episodes of every vigilance state during the periods of light and dark cycle, and a particular β rhythm with the frequency of 20–35 Hz (Jeantet et al., 2013; Lebreton et al., 2015). Promoting sleep with the pharmacological method effectively slowed the decline of cognition performance in HD mice (Pallier et al., 2007). Imposing a drug-induced relative regular rhythm of wake–sleep contributed to the enhancement of cognition ability in HD mice (Pallier and Morton, 2009). In addition, the administration of OX1R and OX2R antagonist significantly attenuated the β activity during the sleep stage of SWS and REM, reduced sleep–wake rhythm deficiency, and efficiently improved the behavioral performance in HD mice (Cabanas et al., 2019; Figure 2A). These results indicate that modulating orexinergic system may have a therapeutic potential for the improvement of sleep and cognitive performance in HD.

Dorsal striatum is the major pathological region in HD (Page et al., 2000). OXA increased the surface levels of α-amino-3-hydroxy-5-methyl-4-isoxazolepropioinc acid (AMPA) receptor in the striatum, leading to the functional changes of striatum circuits (Shin et al., 2009). Both OX1R and OX2R were expressed in dorsal striatum (Zhang et al., 2007). It suggests that orexinergic system may involve in HD pathology by regulating the activity of striatum circuits. Thus, modulating orexinergic system in dorsal striatum-related circuits may be a potential target for the HD treatment.

### Orexinergic System in MS

MS is a kind of long-term inflammation-related autoimmune disease with the major characteristic of demyelinating in the central nervous system, ultimately leading to the neurodegeneration (Correale et al., 2017; Gencer et al., 2019). As the progress of MS is variable, MS is classified into relapsing remitting MS (RRMS), secondary progressive MS (SPMS), primary progressive form of MS (PPMS), and progressive relapsing form of MS (PRMS) (Lublin and Reingold, 1996; Correale et al., 2017; Hughes et al., 2018). The major characteristic of patients with RRMS is experiencing partial or complete recovery from the emergence of recurring symptoms of the disease (Antel et al., 2012; Correale et al., 2017). After periods of years, some of the patients slowly turn into gradual deterioration, the stage called SPMS (Antel et al., 2012; Correale et al., 2017). About 15% of the patients present a progressive decline of neurological function without obvious relapses, which is called PPMS (Antel et al., 2012; Correale et al., 2017). A group of patients with MS who undergo episodes of distinct clinical relapse are taken as PRMS (Hughes et al., 2018). Although environmental element, inflammation, and genetic factors are implicated in MS pathology, the precise mechanisms of MS remain unclear (Correale et al., 2017).

Sleep disturbance is a trigger of relapse in MS, indicating the potential roles of the orexinergic pathway in MS (Sahraian et al., 2017). A significant decrease of OXA in CSF was observed in a female MS patient with hypersomnia (Oka et al., 2004). However, Constantinescu et al. showed that there was no significant deficiency of OXA in patients with MS compared with controls with other inflammatory-related brain disorders or non-inflammatory brain diseases (Constantinescu et al., 2011; Burfeind et al., 2016). A recent study showed that serum OXA was remarkably decreased in patients with MS compared with that in healthy cases (Gencer et al., 2019). Moreover, serum OXA was robustly lower in patients with SPMS than that in patients with RRMS, indicating the serum OXA may correlate with the progression of MS (Gencer et al., 2019). Given the complexity and variety of the pathological courses of MS, further investigation is necessary to determine the orexin levels in CSF of patients with MS at different courses (Figure 2B).

Evidence indicates that neuroinflammation and glial activation are the important pathological features of MS (Datta et al., 2017; Grajchen et al., 2018). Experimental autoimmune encephalomyelitis (EAE) was a commonly used animal model of MS (Becquet et al., 2019). OXA significantly reduced the formation of reactive oxygen species (ROS) in primary cultured microglia (Tunisi et al., 2019; Figure 2B). Moreover, the intraperitoneal injection of OXA decreased lipopolysaccharide (LPS)-induced activation of microglia in the prefrontal cortex, which was blocked by OX1R antagonist SB334867 (Tunisi et al., 2019), indicating the regulation role of the orexinergic system in neuroinflammation (Figure 2B). In addition, the OXA treatment significantly reduced the inflammation and decreased the pathological scores in EAE mice, which was inhibited by SB334867 (Fatemi et al., 2016; Figure 2B). Consistently, the administration of OXA dramatically reduced clinical symptoms, decreased neuroinflammation, and alleviated demyelinating and glial activation in EAE mice (Becquet et al., 2019; Figure 2B). All the above studies indicate that the orexinergic system exerts the neuroprotection roles by inhibiting the neuroinflammation in MS (Becquet et al., 2019).

## Perspective of Targeting Orexinergic System

Given the dysfunction of orexinergic pathway is implicated in the pathogenesis of AD, PD, HD, and MS, the orexinergic system may be a potential therapeutic target for the treatment of these diseases (Petersen et al., 2005; Asai et al., 2009; Fatemi et al., 2016; Mander et al., 2016; Liguori, 2017). Dysfunction of sleep–wake, abnormal protein aggregation, and neuroinflammation are the common features of these diseases (Glass et al., 2010; Correale et al., 2017; Illarioshkin et al., 2018). First, dysregulation of the orexinergic system is implicated in the disruption of the sleep–wake process in AD, PD, HD, and MS (Asai et al., 2009; Mander et al., 2016; Liguori, 2017; Sahraian et al., 2017; Cabanas et al., 2019; Turkoglu et al., 2020). On the one hand, sleep disruption contributes to neurodegeneration by facilitating disease-associated protein accumulation such as Aβ (Mander et al., 2016; Liguori, 2017). On the other hand, sleep disruption also attenuates the clearance of Aβ, whereas sleeping facilitates Aβ clearance in AD model mice (Xie et al., 2013). Moreover, abnormal protein aggregation-induced neuroinflammation aggravates the neurodegeneration and neuronal loss in AD and PD (Glass et al., 2010). Thus, targeting the orexinergic system might ameliorate sleep impairment attenuating abnormal protein aggregation and subsequent neuroinflammation and neurodegeneration.

## Conclusion

Mounting evidence shows that the orexinergic system is involved in the sleep–wake process, whereas sleep disruption is a common symptom of many neurodegenerative diseases including AD, PD, HD, and MS. Moreover, dysfunction of the orexinergic pathway is implicated in the pathogenesis of AD, PD, HD, and MS, which is associated with sleep impairment, pathogenic protein aggregation, neuronal loss, and activation of neuroinflammation (Figures 1, 2). However, the exact roles of the orexinergic system in the above diseases and underlying mechanisms are still not fully understood. Thus, it is essential to further investigate the dysregulation of the orexinergic system in neurodegenerative diseases and its role in the pathogenesis of these diseases.

## Author Contributions

QW and FC wrote the manuscript. YW conceived and revised the manuscript. All the authors approved the final manuscript.

## Conflict of Interest

The authors declare that the research was conducted in the absence of any commercial or financial relationships that could be construed as a potential conflict of interest.

## Publisher’s Note

All claims expressed in this article are solely those of the authors and do not necessarily represent those of their affiliated organizations, or those of the publisher, the editors and the reviewers. Any product that may be evaluated in this article, or claim that may be made by its manufacturer, is not guaranteed or endorsed by the publisher.
